# Microvessel prediction in H&E Stained Pathology Images using fully convolutional neural networks

**DOI:** 10.1186/s12859-018-2055-z

**Published:** 2018-02-27

**Authors:** Faliu Yi, Lin Yang, Shidan Wang, Lei Guo, Chenglong Huang, Yang Xie, Guanghua Xiao

**Affiliations:** 1 Quantitative Biomedical Research Center, Department of Clinical Sciences, University of Texas Southwestern Medical Center, 5325 Harry Hines Blvd, Dallas, TX 75390 USA; 2 Department of Pathology, National Cancer Center/Cancer Hospital, Chinese Academy of Medical Science and Peking Union Medical College, No. 17 Panjiayuan Nanli, Chaoyang District, Beijing, 100023 P. R. China; 3 Department of Bioinformatics, University of Texas Southwestern Medical Center, 5325 Harry Hines Blvd, Dallas, TX 75390 USA; 4 Harold C. Simmons Comprehensive Cancer Center, University of Texas Southwestern Medical Center, 5325 Harry Hines Blvd, Dallas, TX 75390 USA

**Keywords:** Pathology image, H&E images, Angiogenesis, Microvessel, Fully convolutional neural networks

## Abstract

**Background:**

Pathological angiogenesis has been identified in many malignancies as a potential prognostic factor and target for therapy. In most cases, angiogenic analysis is based on the measurement of microvessel density (MVD) detected by immunostaining of CD31 or CD34. However, most retrievable public data is generally composed of Hematoxylin and Eosin (H&E)-stained pathology images, for which is difficult to get the corresponding immunohistochemistry images. The role of microvessels in H&E stained images has not been widely studied due to their complexity and heterogeneity. Furthermore, identifying microvessels manually for study is a labor-intensive task for pathologists, with high inter- and intra-observer variation. Therefore, it is important to develop automated microvessel-detection algorithms in H&E stained pathology images for clinical association analysis.

**Results:**

In this paper, we propose a microvessel prediction method using fully convolutional neural networks. The feasibility of our proposed algorithm is demonstrated through experimental results on H&E stained images. Furthermore, the identified microvessel features were significantly associated with the patient clinical outcomes.

**Conclusions:**

This is the first study to develop an algorithm for automated microvessel detection in H&E stained pathology images.

## Background

The tumor microenvironment includes tumor cells, the blood and lymphatic vasculatures, stroma, nerves, and cells of the immune system [1]. Currently, many studies are focusing on the interactions of tumor cells and immune cells due to the emerging significance of immunotherapy. Moreover, tumor vasculatures have also long been a therapeutic target of anti-angiogenesis [2]. Angiogenesis refers to the formation of new blood vessels from the endothelium of the existing vasculature. Some anticancer medicines aim to cut down the growth of micro blood vessels in order to kill tumor cells or make ill-formed vessels into normal ones (vessel normalization) to channel anticancer medicine into tumor cells and kill them [3]. Thus, it is essential to explore the role of micro blood vessels in the tumor micro- environment.

Microvessel density (MVD) is commonly used as a surrogate measure for angiogenesis. Many studies have shown that MVD is an important prognostic factor in various types of cancers, including lung, breast, colon, cervix, melanoma, and head and neck cancers [4–11]. More importantly, MVD could be a potential indicative factor for predicting chemotherapy response. Furthermore, a very recent study showed that the mechanisms of vessel normalization are correlated with immunotherapy response [12]. Therefore, it is important to quantify the fine architectural features of micro vessels and investigate their role in tumor progression and treatment response. Finally, a convenient and accurate measure of MVD before treatment could serve as a potential biomarker for personalized treatment for individual patients. Although in clinical pathology practice it is not difficult to detect micro blood vessels under microscopic observation, it is hard to quantify MVD by the naked eye. Recent studies have developed computerized algorithms to extract tumor morphological features from H&E slides, and correlate these features with patient outcomes for breast cancer [13, 14] and lung cancer [15, 16]. In this paper, we aim to develop computerized algorithms to automatically detect micro blood vessels in H&E stained image slides, and to study the association between MVD and patient outcomes.

In biomedical research, immunohistochemistry (IHC) staining of cluster determinant 31 (CD31) or 34 (CD34) are the most commonly used methods to identify microvessels in tissue slides [17, 18]. In CD31/CD34 stained images, microvessels appear in a specific color, depending on the stain used (e.g., brown with DAB). These slides are then examined by pathologists. Moreover, because CD31/CD34 staining is specific to studying microvessels, IHC-stained images are rarely available in any existing public datasets, such as The Cancer Genome Atlas (TCGA), which greatly hinders research into the role of microvessels in tumor progression and response to treatment.

**Fig. 1:**
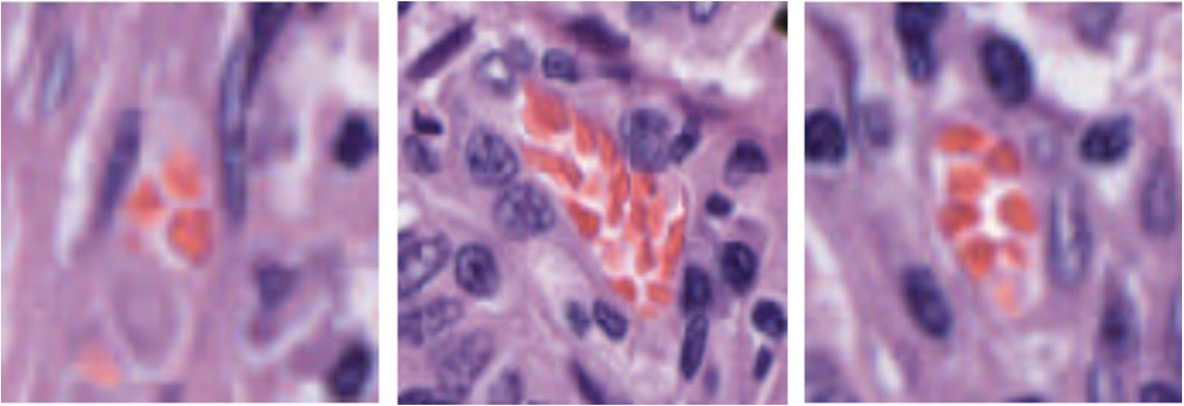
Illustration of microvessels in H&E-stained histopathological images

Hematoxylin and eosin (H&E) staining images are widely used by pathologists. The hematoxylin stains nuclei in a dark blue color and eosin stains other structures as a pink color [19–22]. The H&E-stained images can facilitate morphological feature analysis derived from cell nuclei. Several studies have shown that the H&E-stained image features could predict patient outcome in different types of cancers [23–26]. There are many H&E-stained pathology images in public databases such as TCGA and the National Lung Screening Trial (NLST). Some microvessels shown in H&E-stained images from the TCGA dataset are illustrated in Fig. 1. However, manually identifying microvessels by a pathologist is a labor-intensive and subjective task because of the complexity and heterogeneity of microvessels’ appearance in H&E-stained histopathology images. Therefore, it is important to develop automated microvessel identification methods based on H&E-stained pathological images. At present, however, there is no current research on detecting microvessels from H&E-stained images.

In recent years, deep learning has shown its power in image processing and computer vision with the advent of big data, parallel computing and optimization algorithms [27–29]. Deep learning algorithms such as convolutional neural networks (CNN), fully convolutional neural networks (FCN), and region-based convolutional neural networks have been widely used in image segmentation, object classification and recognition, tracking and annotation [30–41]. To some extent, the prediction results from deep learning algorithms have better accuracy than human predictions [27–29]. In comparison, traditional machine learning algorithms trained based on handcrafted features are designed by humans and require some prior knowledge on the specific problem. Deep learning algorithms can build the features and select the discriminating feature set using a data-driven method. Deep learning methods have achieved promising results on complex data such as image, voice and text, where handcrafted features are not easily defined for high-level analyses such as segmentation, recognition, and classification. In this study, we aim to develop an automated method for microvessel detection in H&E-stained histopathology images using an FCN technique, which is an end-to-end image training method. It is also expected that some other recently developed deep learning algorithms such as U-Net [39], SegNet [40] and fully convolutional DenseNets [41] that are based on FCNs can be applied to microvessel analysis. In this study, a pathologist manually labelled some microvessels in H&E-stained images, which were then used as a training set. All the labelled microvessels were also checked and agreed upon by a second pathologist. Then, an FCN was trained with its parameters initialized from these values in a pre-trained deep learning model. Finally, the fine-tuned FCN was applied to detect the microvessels in a new set of H&E-stained images. To the best of our knowledge, this is the first study to detect microvessels in H&E-stained pathology images using a deep learning algorithm. Experimental results have shown the feasibility of the proposed algorithm. The paper is organized as follows: In Section II, we present the proposed algorithm for microvessel prediction. In Section III, the experimental results are shown. In Section IV, the conclusions and future work are discussed.

## Methods

FCNs are evolved from CNNs [31] and have been the mainstream approach in the field of semantic segmentation since good performance was achieved in [34]. The FCN algorithm can produce end-to-end image training and achieve pixel-wise prediction. Different from other deep learning algorithms such as CNNs, the input image size for FCNs can be arbitrary. FCNs have been widely applied to biomedical images such as MRIs and CT scans, with promising results [42–45]. Many new approaches based on FCNs in specific scenarios have been also proposed and studied in image segmentation, classification, and tracking [39–41, 46–48].

**Fig. 2:**
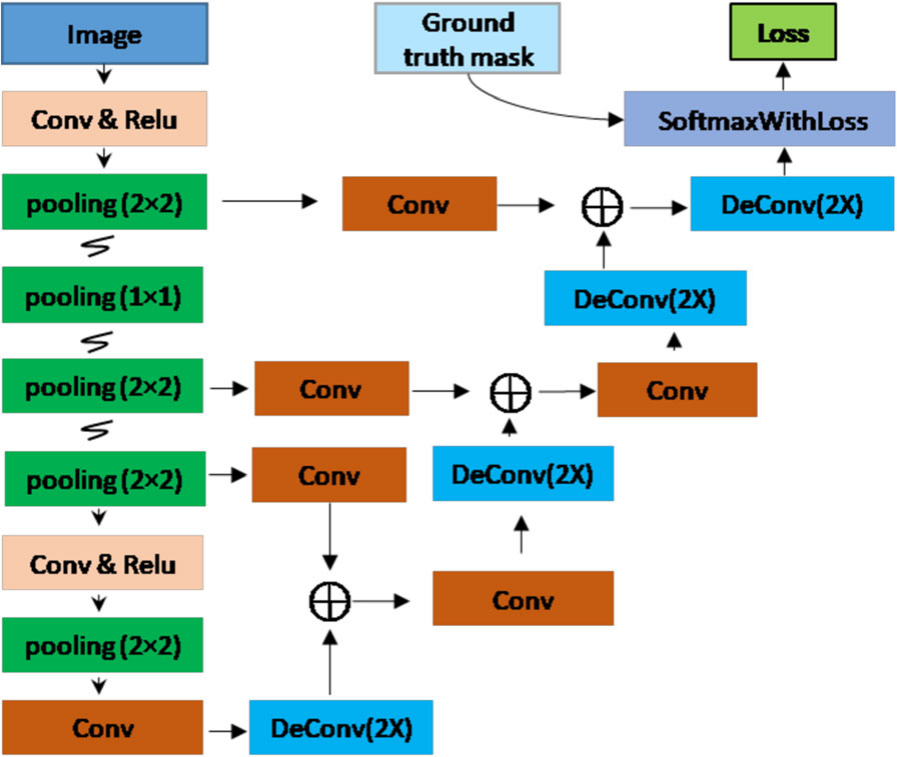
The FCN structure used in this study

FCNs are a specific type of CNN using only size-agnostic layers (e.g. convolutional, pooling). The network architecture of the FCNs used in this study is shown in Fig. 2. This network is constructed with five basic layers, which are Convolution (Conv), Pooling (pool), Rectified linear units (Relu), Deconvolution (deConv), and SoftmaxWithLoss [27, 34]. The convolution layer [46] refers to the convolution operation between image (feature map) and kernel (filter) that is expressed as the following equation:

1 $$ output\left[x,y\right]= input\left[x,y\right]\otimes kernel\left[a,b\right]={\sum}_{b=0}^{columns-1}{\sum}_{a=0}^{rows-1} input\left(x-a,y-b\right) kernal\left(a,b\right) $$

where *input[x, y]* denotes the input image or feature maps within the network, *kernel[a, b]* represents the filter, *rows* and *columns* mean the size of the kernel (filter) in vertical and horizontal rows respectively, and *output[x, y]* is the output of the convolution operation. Neurons of a given image or feature map share their weights on kernels but have different input fields. The pooling [46] operation in this FCN mainly refers to max pooling that simply takes a *k* × *k* region and outputs a single value, which is the maximum value in that region. Max-pooling has the advantage of leading to a faster convergence rate by selecting superior invariant features that can improve generalization performance. Relu [46] is an activation function that brings non-linearity into networks and has been widely used in deep learning algorithms in the last few years. Compared with other common activation functions that involve expensive operations, such as sigmoid and tanh [49, 50], Relu can be implemented by simply thresholding a matrix of activations at zero. Moreover, it is reported that Relu can greatly accelerate the convergence of stochastic gradient descent compared to the sigmoid or tanh functions [49, 50]. Deconvolution [46] is simply viewed as the combination of up-pooling and convolution. Similar to a normal convolutional layer, the kernel used in a deconvolutional layer is learned during the training step. The deconvolutional layer in an FCN algorithm is mainly used to make the size of the output image the same as that of the input image. Consequently, the FCN algorithm can handle images with arbitrary sizes during both training and prediction steps. The SoftmaxWithLoss layer [46] computes the multinomial logistic loss for a one-of-many classification task, passing real-valued predictions through a softmax [46, 49, 50] to get a probability distribution over classes. This layer is fundamental to the training phase, because the loss function contributes to the update of network parameters. The SoftmaxWithLoss layer is the combination of softmax and multinomial logistic loss. All parameters used in FCN are learned during the training phase by minimizing the loss function using the backpropagation algorithm [49, 50]. During the testing phase, the SoftmaxWithLoss layer can be replaced by a Softmax Layer [46].

The FCN structure of this study in Fig. 2 is different from that used in [34]. There are only four max pooling layers (Although there are five boxes denoting max pooling in Fig. 2, one is operated within a 1 × 1 region, which means nothing has been done) in our FCN structure, and the last two convolutional layers in [34] (the fully connected layers used in CNN) were not included in order to reduce the length of the networks. Therefore, the efficacy in learning and inference can be improved. Moreover, the prediction results in this FCN structure are only 2**×** upsampled from the previous layers, which have fused information from all max pooling layers, while the prediction results in [34] are at least 8× upsampled from previous layers that have more coarse values.

In this study, we developed the model using a training set (with 300 images at a size of 384 × 384 pixels, extracted from 10 H&E pathology slides), a validation set (with 50 images at a size of 384 × 384 pixels, extracted from another 5 slides) and a testing set (with 35 large images at a size of 1600 × 1600 pixels, extracted derived from 5 new slides). All the image slides were derived from different patients. The training images were first normalized with a standardization method [46] based on the population statistics of the training dataset. In this study, we initialized all weights in the algorithm with a pre-trained neural network and then fine-tuned the weights using images from the training set. In order to prevent the algorithm from over-fitting, we used a validation set to determine the iteration number and a stopping criterion. Then, the prediction performances of the model were evaluated in the testing set. After the model was developed, we applied the model to identify the micro vessels in the pathology images of 88 lung adenocarcinoma patients from the Chinese Academy of Medical Sciences (CHCAMS) cohort to study the association between MVD and patient survival outcomes (see Fig. 4 for more details).

## Results

In this paper, all of the results were obtained from the computer experiment using the interface of Python 3.0 based on a Caffe deep learning framework [51], which was installed and executed on a server with Linux version 3.16.0-69-generic and Ubuntu 4.8.2-19 in 64 bits. This server also includes two Intel(R) Xeon(R) CPU E5-2680 v3 processors of 2.50 GHz and a 30 Mb Cache, where each processor has 12 cores and the total number of logical CPU cores is 48. The server has 132 Gb RAM and an NVIDIA Tesla K40 m GPU with 2880 stream cores, 12 Gb maximum memory, 288 Gigabytes/s maximum memory bandwidth, and 6GHz memory clock speed.

**Fig. 3:**
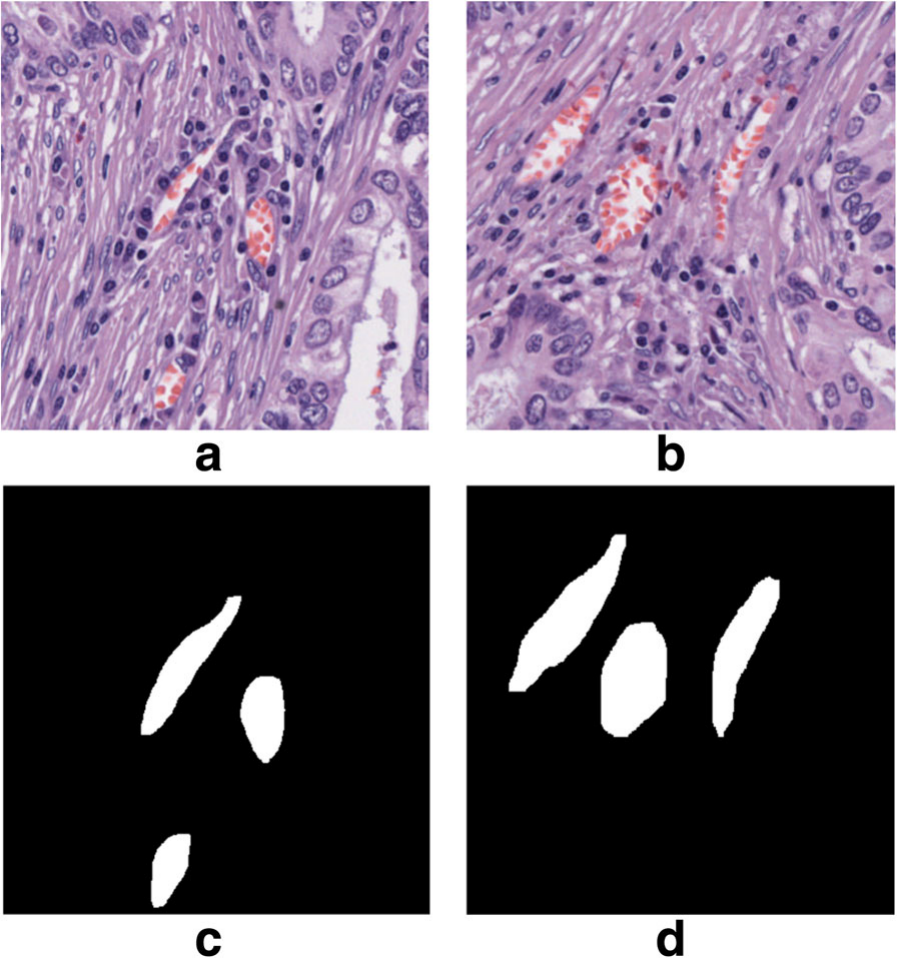
Illustration of two H&E stained images with microvessels labelled. **a** & **b** two H&E stained images. **c** & **d** the corresponding microvessel masks of images in (**a**) & (**b**)

**Fig. 4:**
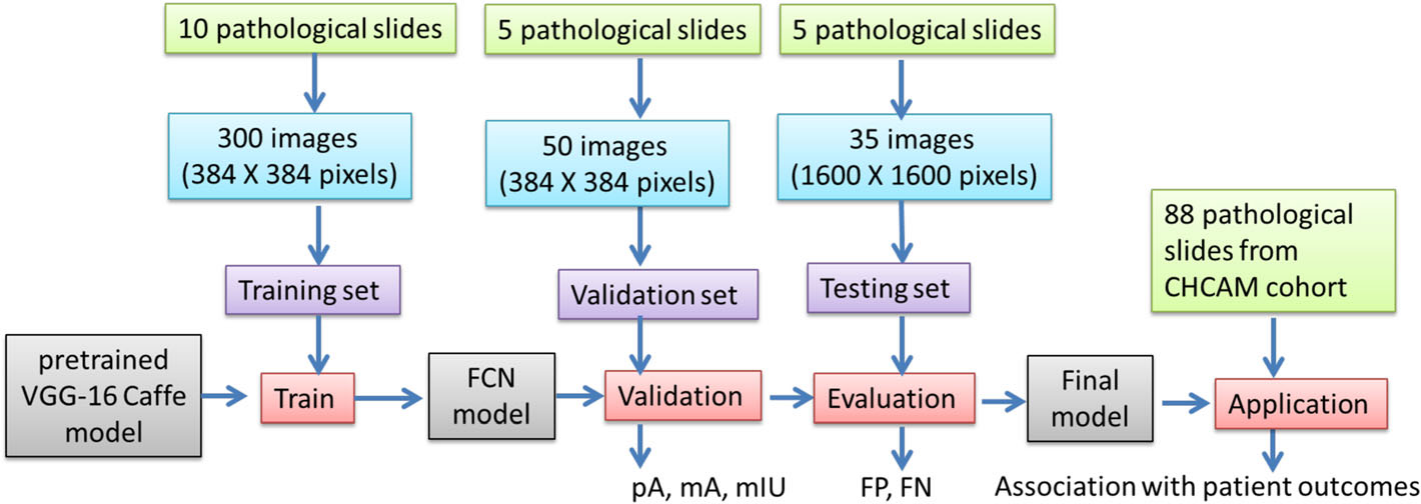
Flowchart of the training, testing and evaluation strategy

The H&E stained histology images for lung adenocarcinoma (ADC) patients were from the National Cancer Center/Cancer Hospital, CHCAMS and Peking Union Medical College, China. These slide images were at 20X magnification with a resolution of 0.5 μm/pixel. 300 images at a size of 384 × 384 (pixels) were extracted from 10 H&E stained pathology slide images and were used for algorithm training. Another 50 images at a size of 384 × 384 (pixels) were extracted from 5 new slides and used as a validation dataset. In order to improve the accuracy of the manually labelled microvessels, we had two pathologists, Drs. Lei Guo and Lin Yang, label the blood vessels independently, and used the blood vessels that both pathologists agreed on as the ground truth for evaluating the performance of the algorithms (the overall agreement between the two pathologists was around 85%). The pixels within the microvessels were labelled as foreground (with value equals to 1) while other regions were denoted as 0 s. All these labelled microvessels were checked and agreed on by another pathologist. Two exemplary H&E stained images with labelled microvessels are shown in Fig. 3. Finally, 35 large images with size of 1600 × 1600 (pixels) were extracted from 5 new H&E stained pathology slide images and used as testing images to evaluate the performance. The training, testing and evaluation strategy is summarized in Fig. 4.

The FCN structure was developed using a Caffe [51] framework and conducted on a GPU. Some layers of the FCN structure from this study are similar to those in the VGG-16 networks [52], and some layers are not. The parameters of the layers of this network that were the same as the VGG-16 networks were initiated from a pre-trained VGG-16 Caffe model, while the parameters in layers that were different from VGG-16 networks were initialized with a Xavier algorithm [53]. Then, this FCN network was fine-tuned using the training images while the size of min-batch was set as 5. For the FCN training, a stochastic gradient descent algorithm was applied to optimize the loss function in order to fine-tune the FCN model. The momentum value was given as 0.99 and the weight decay, which is used to regularize the loss function, was set as 0.0005. Further, the learning rate was initialized as 0.01 and decreased by a factor of 10 every 1000 iterations.

**Fig. 5:**
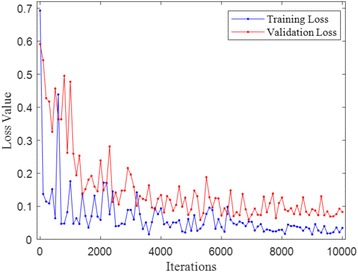
Loss values during FCN training

**Fig. 6:**
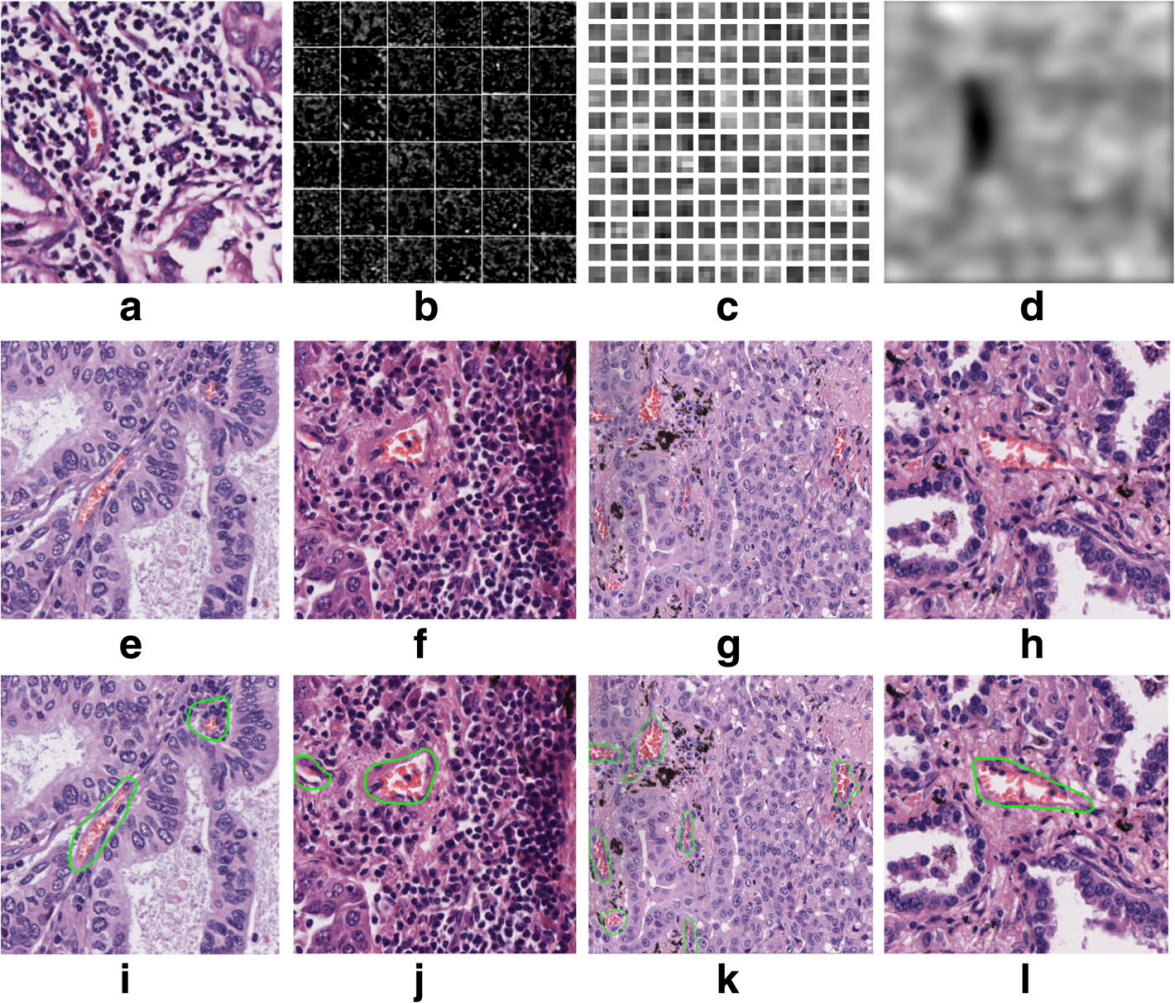
Illustration of feature maps, and prediction results learned weights. **a** An H&E stained image. **b** Some features maps in the convolution layer. **c** Some learned weights in the convolution layer. **d** One feature map in the deconvolution layer. **e**-**h**: Original H&E stained images. **i**-**l** Corresponding microvessel detection results from trained FCN model

The loss values during the FCN training phase based on the training and validation datasets are measured and shown in Fig. 5. As shown in Fig. 5, there were small fluctuations for the loss values in the training dataset, and it tended to be more stable when the iteration number approached 6000. This indicated that the FCN structure had learned the microvessel features. Some of the feature maps and learned parameters are presented in Fig. 6 (a) – (d). It is noted from Fig. 6(h) that the microvessel areas have much different information than the non-microvessel areas. After the training phase, the trained FCN model was then used to detect the microvessels in new input H&E stained images. Some of the detection results from the FCN model are shown in Fig. 6 (e) – (i). It shows that most of the microvessels were successfully detected.

Moreover, the microvessel segmentation results were quantitatively measured based on 50 H&E stained 384 × 384 pixels testing images, which were not used in the FCN training phase. The metrics consisting of pixel accuracy (pA), mean accuracy (mA), and mean intersection over union (mIU) were adopted for the segmentation evaluation. These metrics were defined as the following:

2 $$ pA={\sum}_i{n}_{ii}/{\sum}_i{t}_i $$

3 $$ mA=\left(\frac{1}{n_{cl}}\right){\sum}_i{n}_{ii}/{t}_i $$

4 $$ mIU=\left(1/{n}_{cl}\right)\ {\sum}_i{n}_{ii}/\left({t}_i+{\sum}_j{n}_{ji}-{n}_{ii}\right) $$

where *n*_*ij*_ is the number of pixels of class *i* predicted to belong to class *j*, *n*_*cl*_ is the total number of different classes, and *t*_*i*_=∑_*j*_*n*_*ij*_ is the total number of pixels of class *i*. In addition to the aforementioned metrics, numbers of false positive (FP) and false negative (FN) results were used for the segmentation evaluation. FP refers to the number of predicted microvessels that were not actually microvessels, and FN means the number of microvessels that were not successfully detected. A total of 35 pathology images of size 1600 × 1600 were extracted from another 5 H&E stained slides, which were not used in the FCN training and testing phase (see Fig. 4). The trained FCN was applied to these new 35 images in order to evaluate FP and FN. The total number of microvessels in these images was about 450. In this study, the FCN-8 s model proposed in [34] was also applied to detect the microvessel and used for comparison. The prediction evaluation results of both our FCN model and FCN-8 s are shown in Table 1. It is noted from Table 1 that the proposed FCN model outperforms the FNC-8 in all defined metrics. The pathologist checked the FPs and found the two models seem to mis-classify regions having blood cells as microvessels, but in fact the appearance of blood cells doesn’t guarantee a microvessel is present. It is expected that the FP problem could be reduced with more training images, which contain more non-microvessel areas with some blood cells.

**Table 1:** Prediction results between our FCN and FCN-8 s

	The proposed FCN model	FCN-8 s
pA	0.952	0.946
mA	0.833	0.772
mIU	0.755	0.707
FP	119	155
FN	7	22

In addition to the aforementioned metrics, the training time and prediction/inference time were measured and compared between our FCN model and FCN-8 s in Table 2. This indicates that the proposed FCN model consumes less inference time than FCN-8 s in [34], while more training time is needed.

**Table 2:** Time consumption between our FCN and FCN-8 s

	The proposed FCN model	FCN-8 s
Training time [ms]^a^	1.2E + 07	1.2E + 07
Inference time [ms]^b^	313	390

^a^measured based on 300 training images of size 384 × 384 and the total iteration is 10,000

^b^measured based on 50 validation images of size 384 × 384

Since it is difficult to gather a large number of labelled images for FCN training from scratch, fine-tuning the networks is a good strategy using a limited number of training images. In total, the proposed FCN model has fewer layers than that used in FCN-8 s, and it makes good use of the limited training images and leads to less inference time. However, one more fusion layer used in our model may make the back-propagation more complicated and require a longer training time. The prediction layer in FCN-8 s is 8X upsampled from the previous layer and it produces more coarse results compared with that in our FCN model, where the prediction result is only 2X upsampled from the previous layers and thus can improve precision. It is also expected that the prediction accuracy for both FCN models could be improved if more training images were provided.

In this study, we applied the trained FCN model to identify the microvessels in the pathology images of 88 lung adenocarcinoma patients from the CHCAMS cohort. First, a lung cancer pathologist identified and labeled the tumor region(s) from each tissue slide in agreement with another pathologist, and then we randomly sampled three representative images from each tumor region. The total number of sample images collected was 274. For each sample image, we identified the microvessels using the FCN model. Then, we calculated the total microvessel area in each image, as well as the percentage of tumor cells around the microvessel, defined by the number of tumor cells around the microvessel divided by the total number of cells around the microvessel.

With the estimated total area of microvessels and the percentage of tumor cells around the microvessel, we fit a Cox regression model to evaluate the association between estimated microvessel features and patient survival outcomes, after adjusting for other clinical information such as age, gender, and tobacco history. Multiple sample images from the same patient were modelled as correlated observations in the Cox regression model to compute a robust variance for each coefficient. The hazard ratio (HR), the 95% confidence interval (CI) of the HR and the *p*-value for each variable are summarized in Table 3.

**Table 3:** Survival analysis for NLST lung cancer pathology images

	HR (95% CI)	*p*-value
Gender (Male vs. Female)	1.05 (0.40, 2.75)	0.922
Age	1.01 (0.98, 1.04)	0.433
Tobacco history (Yes vs. No)	1.15 (0.44, 3.01)	0.779
microvessel Area	0.35 (0.14, 0.90)	0.029*
Percentage of Tumor Cells	3.14 (0.88, 11.24)	0.078

The results show that higher microvessel density in a patient is associated with significantly better survival outcome. This finding is consistent with current studies in lung cancer [3] and kidney cancer [54]. In addition, a higher percentage of tumor cells around the microvessels is associated with poor survival outcome, but the *p*-value is only marginally significant, probably due to the limited sample size (*n* = 88).

**Fig. 7:**
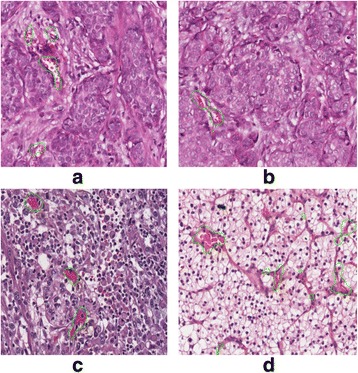
microvessel prediction results in H&E-stained image with breast cancer (**a**) & (**b**) and kidney cancer (**c**) & (**d**)

Furthermore, we applied the developed algorithm to other types of H&E studies. Some microvessel prediction results based on H&E-stained images in breast and kidney cancers are shown in Fig. 7. The current algorithm seems to perform reasonably well for H&E-stained images in different types of cancer. However, a systematic evaluation is needed for further study.

## Discussion

In this paper, we propose a deep learning algorithm to detect microvessels in H&E stained pathology image. Experimental results verified that the features of complex microvessels could be learned and used for microvessel detection using FCN models. Furthermore, these microvessel prediction results were evaluated and validated by a pathologist. Although the training phase in FCN takes a relatively long time, the computing time in the prediction/inference phase is acceptable. Comparison results have shown that our proposed method produces better results than the original FCN-8 in terms of pixel accuracy, mean accuracy, mean intersection over union, FP, FN, and inference time. This study developed a computer algorithm to detect micro blood vessel and quality micro blood vessel related features, such as MVD, from the H&E stained images. It provides an alternative way to study the role of micro blood vessels and investigate their role in tumor progression and treatment response from public datasets, when the CD31/CD34 IHC stained images are not available.

In this study, we used the manually labelled microvessels within the H&E-stained images as the ground truth for the measurement of algorithm performance. In order to improve the accuracy of the manually labelled microvessels, we had two pathologists, Drs. Lei Guo and Lin Yang, label the blood vessels independently. We noticed that the inter-observer variability was relatively high, especially for relatively small blood vessels. So, only the blood vessels that both pathologists agreed on were used as the ground truth for evaluating the performance of the algorithms. Moreover, the model developed from this study provides an objective method for micro blood vessel detection from H&E stained images for future studies and clinical applications.

In this study, we used a training set to train the model, and an external validation set to determine the numbers of iterations in order to avoid overfitting. Next, we evaluated the prediction performance of the final model in the testing set. The final model was applied to a new cohort (88 lung adenocarcinoma patients from the CHCAMS cohort) to identify micro blood vessels and study the association between the micro blood vessel-related features and patient outcomes, while the underlying biological mechanisms merit further investigation.

In this study, we developed a deep learning-based algorithm for detecting micro blood vessels from H&E stained images, mainly from lung cancer. The proposed method could also be applied to other types of cancers, such as breast and kidney cancers. Currently, the proposed FCN model algorithm may have a false positive problem for background regions where a large number of blood cells appear. These problems could be resolved by feeding the algorithm with more training data, including a greater variety of microvessels and non-microvessels.

## Conclusions

It has been reported that microvessel-based features in immunochemistry images are potentially associated with patient outcome. To the best of our knowledge, there is no related research on microvessels in H&E stained images. In this study, the proposed method was used to identify microvessels in a real patient cohort, and the resulting microvessel density is significantly associated with patient survival outcome. This indicates that our method has the potential to predict patient clinical outcome using H&E pathology images, which are widely available in clinical practice.
